# Deliberating Inequality: A Blueprint for Studying the Social Formation of Beliefs about Economic Inequality

**DOI:** 10.1007/s11211-022-00389-0

**Published:** 2022-04-01

**Authors:** Kate Summers, Fabien Accominotti, Tania Burchardt, Katharina Hecht, Elizabeth Mann, Jonathan Mijs

**Affiliations:** 1 London School of Economics and Political Science, Houghton Street, London, WC2A 2AE UK; 2 University of Wisconsin-Madison, Madison, USA; 3 University of Konstanz, Konstanz, Germany; 4 Boston University, Boston, USA; 5 Erasmus University Rotterdam, Rotterdam, Netherlands

**Keywords:** Economic inequality, Perceptions, Public opinion, Deliberative focus groups, Experimental methods

## Abstract

In most contemporary societies, people underestimate the extent of economic inequality, resulting in lower support for taxation and redistribution than might be expressed by better informed citizens. We still know little, however, about how understandings of inequality arise, and therefore about where perceptions and misperceptions of it might come from. This methodological article takes one step toward filling this gap by developing a research design—a blueprint—to study how people’s understandings of wealth and income inequality develop through social interaction. Our approach combines insights from recent scholarship highlighting the socially situated character of inequality beliefs with those of survey experimental work testing how information about inequality changes people’s understandings of it. Specifically, we propose to use deliberative focus groups to approximate the interactional contexts in which individuals process information and form beliefs in social life. Leveraging an experimental methodology, our design then varies the social makeup of deliberative groups, as well as the information about inequality we share with participants, to explore how different types of social environments and information shape people’s understandings of economic inequality. This should let us test, in particular, whether the low socioeconomic diversity of people’s discussion and interaction networks relates to their tendency to underestimate inequality, and whether beliefs about opportunity explain people’s lack of appetite for redistributive policies. In this exploratory article we motivate our methodological apparatus and describe its key features, before reflecting on the findings from a proof-of-concept study conducted in London in the fall of 2019.

**Supplementary Information:**

The online version contains supplementary material available at 10.1007/s11211-022-00389-0.

## Introduction

Rising levels of income and wealth inequality since the 1980s have fueled a robust stream of public opinion research exploring how people *perceive* inequality: How much of it do they think there is? Do they believe this is too much? Where do they suppose inequality comes from? Perceptions in social life often depart from reality, yet investigating their origins is important for understanding how people behave toward that reality. Subjective understandings of inequality’s extent and beliefs about its causes are thus likely to shape people’s preferences for taxation and redistribution in ways that can help curb economic inequality – or in contrast leave it unchecked. In fact, there is strong evidence that people’s attitudes toward inequality track their subjective perceptions of it rather than objectively measured economic disparities (Bartels, 2008; Kuziemko 2015; Niehues, 2014). In this article we propose and pilot a methodological approach for studying how understandings of wealth and income inequality are shaped by social interaction, and how these understandings might respond to different informational stimuli.

Arguably the most solid finding of scholarship on perceptions of inequality is that people are unaware of its extent. While exceptions exist in France, Hungary, or Mexico (Niehues, 2014; Campos-Vazquez et al., 2020; Mijs & Hoy, 2022), a host of survey research shows that in most societies the public underestimates income inequality (Clark & D’Ambrosio, 2015; Engelhardt & Wagener, 2014; Hauser & Norton, 2017; Osberg & Smeeding, 2006). This tendency is especially pronounced in countries that are themselves highly unequal, such as the USA, Chile, South Africa, or the UK (Gimpelson & Treisman, 2018; Norton & Ariely, 2011). In 2009, the average American perceived the ratio of CEOs’ to unskilled workers’ pay in the USA to be around 30:1, which underestimates the actual figure by more than tenfold (Kiatpongsan and Norton 2014).

In an effort to correct these misperceptions and heighten public awareness of economic disparities, researchers have begun fielding survey experiments designed to measure whether the provision of information about inequality might change people’s attitudes toward it and build support for redistributive policies. This approach, which assumes a straightforward relationship between the objective information people receive and the views they form about society, has come with mixed results. McCall and colleagues (2017) thus find that exposing Americans to evidence of rising income inequality increases their skepticism of economic opportunity and hence their support for redistribution. On the other hand, though, Trump (2018) and Heiserman and Simpson (2017) suggest that when income differences in a country are perceived to be high, people come to think of income disparities as more legitimate. Research exposing people to information about their relative position in the income distribution has produced somewhat more consistent findings. For example, Kuziemko and colleagues (2015) show that Americans become more worried about inequality when they realize they are not as economically advantaged as they imagined. Cruces and colleagues (2013) likewise find that poor Argentines grow more supportive of redistribution after learning how poor they are relative to others, while rich Argentines learning how rich they are become less supportive of it (see also Karadja et al., 2017; Mijs & Hoy, 2022).

This article advocates a less haphazard approach. To understand how misperceptions of inequality might be changed, we argue that it is important to start with an investigation of how people form inequality beliefs. Survey approaches are limited in their ability to achieve this, because they cannot fully grasp “the dynamic and contextual aspects of attitude formation” (Zimmermann et al., 2018, p. 969) and “the complexity and potential ambivalence of attitude patterns” (Heuer et al., 2018, p. 97). We further observe that while existing survey experimental research takes *individual participants* as its unit of analysis, in real life people process information and form beliefs about the social world in *social interaction*: by talking with friends, arguing with family members or exchanging views with coworkers. Beliefs may also form in day-to-day interactions with people beyond one’s social circle, for example on public transport, in restaurants, shops or one’s workplace. This means that a realistic account of the formation of inequality perceptions should make room for the role of discussion and interaction networks in shaping people’s understandings of inequality. In this methodological article, we take one step toward building such an account by introducing a research instrument for examining how people talk, process information, and form perceptions about inequality in interactive environments.

### Toward an Account of the Social Formation of Inequality Beliefs

Our approach builds on a broader body of scholarship that stresses the situated character of people’s inequality perceptions, and the interplay between personal biographical circumstances and the recognition of wider structural conditions (Bottero, 2019; Dawtry 2015, 2019; Irwin, 2015, 2016). Research suggests that besides individual factors such as people’s normative beliefs about fairness, equality, or opportunity (Alesina and La Ferrarra 2005; McCall, 2013), beliefs about poverty, affluence, or economic disparities partially arise from a process of “social sampling” (Dawtry et al., 2015, 2019) whereby individuals make inferences about the social world based on the cues they receive throughout their lives from their direct environment (Kuhn, 2019; Mijs 2018). Lending weight to this idea, perceptions of inequality have been found to relate to neighborhood characteristics (Luttmer, 2001; Minkoff & Lyons, 2019), local levels of inequality (Buttrick & Oishi, 2017), pay setting processes in one’s firm (Hecht, 2021) or personal experiences of fortune, misfortune, advantage, or disadvantage across the lifecourse (Edmiston, 2018; Margalit, 2013; Roth & Wohlfart, 2018) that can also turn inequality into a more or less salient issue in people’s eyes (Bottero, 2019). These findings echo Kelley and Evans’ (1995) proposition that perceptions of social structure are a blend of wider inequalities in society and generalizations people form based on their social environment, networks, and observation of reference groups.

An important implication of this line of thinking is that social sampling may explain people’s observed tendency to underestimate economic inequality. If indeed individuals form inequality beliefs by observing the local contexts they are embedded in, their visions are unlikely to reflect the full range of economic disparities they would find by sampling from society at large (Runciman, 1966). Socioeconomic segregation or isolation, then, might be to blame for people’s inability to grasp the magnitude of inequality in many places. This could further explain why underestimations of inequality are particularly pronounced in more unequal societies, as socioeconomic isolation is likely stronger there (Mijs 2021; Mijs & Roe, 2021).

### Deliberative Focus Groups as a Window into People’s Discussion Networks

Expanding on these insights, we propose to study how discussion and interaction networks shape understandings of inequality through a methodology of deliberative focus groups embedded within a broader experimental design. In political theory, deliberation consists in “the public communication of information relevant to the articulation and resolution of public problems” (Anderson, 2010, p. 98; see also Fishkin, 1991, Gutmann & Thompson, 1996). In social science, deliberative focus groups are focus groups wherein moderators introduce outside information at certain junctures to feed discussion among participants (Burchardt, 2014). While political theory sometimes views deliberation as carrying greater democratic legitimacy than the more traditional decision procedures of representative democracy (Cohen, 1989), we here explore deliberative processes for their epistemic value. That is, we draw on deliberation for its ability to reveal the social dynamics undergirding the formation of people’s opinions (Nino, 1996). We see these groups as approximating, however imperfectly, the kind of interactive settings within which individuals process information and develop beliefs about inequality in real social life (see Burchardt, 2014; Davis et al., 2020; Heuer et al., 2018; Zimmerman et al., 2018). This closer approximation of the social world, we believe, means that deliberative groups offer a better picture of the development of inequality beliefs than do less situated techniques—such as survey experiments with individual participants.

At the same time, focus groups remain highly controlled environments that we can experimentally manipulate to study the drivers of inequality beliefs. By systematically varying, from one group to the next, the social makeup of focus groups as well as the information we expose their members to, we use deliberative focus groups to explore how different types of social environments and different types of information shape conversation, deliberation, and ultimately belief formation about economic inequality. In particular, we ask: Do views of inequality developed in deliberative groups differ from those reported by isolated individuals? Are socioeconomically diverse groups more likely than homogeneous ones to report accurate perceptions of inequality—suggesting that socioeconomic isolation might be to blame for people’s misperceptions of it? Our instrument also lets us explore the role of information in shaping the social formation of inequality beliefs: Does the provision of information about inequality alter group participants’ understandings of its magnitude, their concerns about it, and their willingness to scale it down through redistributive policies? If so, what *kind* of information has more sway on people’s inequality views: information about inequality’s extent or information about its recent rise, for example? Finally, does the effect of information, and of different kinds of information, vary across social contexts, and what do these variations look like?

Next, we present our research instrument and experimental design in greater detail, highlighting how their features enable us to address each of the theoretical questions above. In a second step, we provide empirical evidence of the effectiveness of our design, based on the findings from a proof-of-concept study we conducted in London in the fall of 2019. While our initial hope was to present a more comprehensive set of results based on data we were scheduled to collect in the spring and fall of 2020, the Covid-19 pandemic that hit Britain around that time derailed this plan. We therefore limit ourselves to the presentation of our research design, and to a brief empirical demonstration of its ability to elicit the kind of social dynamics this project is after when it comes to exploring the formation of inequality beliefs.

### Studying the Social Formation of Inequality Beliefs: An Experimental Design

#### Overview

Our research instrument consists of deliberative focus groups bookended by pre- and post-focus group surveys. Deliberative focus groups are the tool we use to approximate the interactional contexts wherein individuals form beliefs about economic inequality in real social life. The participant pool for our study is selected to be broadly representative of the British adult population, and to include diversity across key demographic characteristics including age, gender, ethnicity, household makeup, and income level.^1^ In line with our experimental approach, participants from this pool are allocated to one of several intersecting treatment conditions: two deliberative focus group conditions (varying the degree of socioeconomic diversity in the makeup of focus groups) and one individual interview condition working as a control; and, for each of these conditions, four different sets of information about inequality that participants receive from moderators. Pre- and post-surveys are designed to *quantitatively* assess how our various treatments succeed or fail to sway people’s perceptions of income and wealth inequality. The content of our focus groups and individual interviews forms the source of our *qualitative* data, which consist of all conversations and interactions among participants, as well as between participants and moderators, recorded in the various conditions. The sections below elaborate on this basic protocol in greater detail, following the sequence in which participants go through it.

#### Pre- and Post-Focus Group Surveys

Our pre-survey is administered to participants immediately ahead of their focus group or individual interview and a longer post-survey follows two weeks afterwards. Bookending our treatment between repeated surveys gives our research its experimental character, while the timing of the second survey allows us to assess whether treatment effects are durable rather than a blip following participation in group discussions (see Online Appendix A for our survey questionnaires).

Both surveys include the same set of items aimed at measuring descriptive and normative beliefs about inequality in order to compare participants’ responses before and after various treatments. To allow for a comparison with extant research, where possible, we draw from the International Social Survey Program (ISSP), General Social Survey (GSS), European Social Survey (ESS), and European Values Study (EVS), as well as from more detailed studies of inequality perceptions and policy attitudes (e.g., Alesina et al., 2018; Kuziemko et al., 2015; McCall, 2013; Osberg & Smeeding, 2006). While most extant surveys concentrate on income inequality, we also include questions about wealth inequality to take seriously the insight that both matter for people’s experiences and normative evaluations of their and others’ resources and positions (Hecht & Summers, 2020; Townsend, 1979). In particular, we have reason to believe that income, relative to wealth, may be understood as “fairer”—because income is more readily linked to the recognition of one’s performance in the labor market (Rowlingson & Connor, 2011).

#### Baseline Deliberative Task

Each of our deliberative focus groups involves eight adult participants—a size suited to the emergence of meaningful interactions among participants, as well as to successfully conducting and feeding back on the interactive task described later in this section. Focus groups are designed to last for about two hours (see our focus group topic guide in Online Appendix B). After participants have individually completed the pre-focus group survey, a discussion begins with group moderators asking what comes to mind when participants hear the words “income” and “wealth.” Following this, moderators share with participants classic definitions of income and wealth found in the academic literature, in order to establish a common set of meanings among group members. Income is defined as: “Money you receive from work, financial assets or real estate, as well as social security and other benefits.” Wealth is defined as: “The amount of assets that someone owns. This can include their house or houses, cars, savings, stocks and shares, investments, and so on.”

In a second step, group moderators facilitate a “baseline” deliberative segment wherein participants collectively explore their descriptive understandings of income and wealth inequality. This segment starts with a discussion broadly aimed at making participants reflect on the *concentration* of income and wealth in contemporary British society, through the use of cues such as: “Thinking specifically about the UK today, how do you think income is spread out between people?” Following this discussion, participants are divided into subgroups of two to three and handed 100 green Lego bricks, which they are told represent all of the *income* in the UK in a given year. Participants are also handed a paper template depicting ten silhouette figures standing in a line, which participants are told represent all people in the UK, arranged from lowest to highest income group. Group moderators then ask each group of participants to work together to divide the Lego bricks among the figures so as to reflect what they collectively believe the distribution of income looks like today. Upon completion of this task, participants are asked to talk through their considerations and reasoning as they completed the task, and to reflect on the distributions other participants came up with. The task is then repeated with the same materials, but this time with the 100 Lego bricks representing all of the *wealth* in the UK in a given year. To conclude this baseline deliberative task, participants are invited to reflect on the causes that might be shaping the distributions they have created, the difference between income and wealth, to consider what the upsides and downsides of these distributions may be, and to share whether they believe it is possible and desirable to change the status quo as far as these distributions are concerned.

This baseline task is meant to measure whether participants arrive at more accurate estimates of existing levels of inequality when thinking about them in more realistic-looking, deliberative, environments than when they do so in isolation (as classic surveys ask them to). The task does not entail the provision to participants of any outside information about economic inequality. It does, however, create an environment of informed participants (1) by clearly defining income and wealth, (2) by making sure participants think about economic inequality in terms of greater or lesser income or wealth concentration, and (3) by asking them to complete a task (the allocation of Lego bricks to various deciles) that physically instantiates, and therefore makes tangible, the otherwise difficult to grasp notion of an income or wealth distribution. In sum, the task places participants in a position of greater competence to express their perceptions of inequality’s extent.

#### Manipulating Information about Inequality

In our focus groups’ third segment, moderators introduce a set of factual information about economic inequality which allows us to observe its effect on the ensuing discussion and on participants’ inequality beliefs as measured by post-surveys. Our materials mirror stimuli used in past research (e.g., Alesina et al., 2018; Gallego, 2016; Kuklinski et al., 2000; Kuziemko et al., 2015; McCall et al., 2017; Trump, 2018). The originality of our design is to study the impact of information in a focus group setting. Focus groups are randomly allocated to one of three informational treatment conditions or to a control condition without information.

1. In the “true distribution” condition (cf. Kuziemko et al., 2015), moderators use the Lego materials from the baseline task to reveal the actual distributions of income and wealth in the UK, based on data from the World Inequality Database and the UK’s Wealth and Assets Survey, respectively.

2. In the “historical data” condition (cf. McCall et al., 2017), moderators draw on these same data to, first, reveal the present-day distribution of income and wealth in the UK and, in a second step, show how these distributions have evolved in recent history. This condition is designed to challenge the sense of ineluctability participants may have when thinking about economic inequality.

3. In the “social mobility” condition (cf. Alesina et al., 2018), moderators first reveal the distributions of income and wealth in the UK. They then use different-color Lego bricks to show the likelihood of individuals from respectively low and high income families to experience downward or upward social mobility based on data from the British Cohort Study (see Blanden et al., 2019). Motivated by studies describing a widespread popular tendency to overestimate social mobility (Alesina et al., 2018; Kraus & Tan, 2015), our aim is to test whether a more sobering view of mobility in the UK makes participants more concerned about inequality.

4. Finally, in the “no information” condition, participants do not receive any external information about inequality (cf. McCall et al., 2017; Mijs & Hoy, 2022). Including this control condition lets us tease out the impact of information from that of merely participating in a deliberative focus group.

#### Manipulating the Social Makeup of Focus Groups

To examine whether and how the composition of people’s discussion networks may shape beliefs about economic inequality, our design randomly assigns participants to a second set of treatment conditions, defined by the greater or lesser social homogeneity of the focus groups they participate in. In the “diverse condition,” focus groups are mixed in terms of participants’ socioeconomic status, which we measure as their annual income. By contrast, in two “homogeneous conditions” all participants of a given focus group hail respectively from the top or from the bottom half of the British income distribution. In all three conditions, focus groups preserve the diversity of our initial participant pool in terms of other key demographics such as gender, age, or race and ethnicity.

The composition of focus groups in our diverse condition is intentionally artificial, and is a poor approximation of the real-life contexts wherein individuals form beliefs about economic inequality. Homogeneous focus groups, on the other hand, are meant to offer a better reflection of the contexts people typically encounter in their everyday working lives and social networks. Comparing diverse and homogeneous conditions therefore enables us to explore whether artificial groups that are more representative of the whole population come up with better estimates of existing levels of inequality (in the Lego bricks task), as well as with different normative evaluations of what should be done about it (in group discussions), than do more realistic, socioeconomically segregated ones. This offers a direct test of the idea that socioeconomic isolation, and the resulting inability to grasp the full range of economic conditions in the larger population, are to blame for people’s tendency to underestimate inequality (Dawtry et al., 2015; Mijs 2021).

#### Individual Condition

Finally, we randomly assign a number of participants to an individual, interview-like condition in which they do not participate in a focus group but instead go through the steps of our topic guide (definition of income and wealth, Lego bricks task, and exposure to evidence about inequality) alone with a moderator. For the sake of cost effectiveness, participants to this individual condition are only exposed to two informational treatments: the revelation of the true distributions of income and wealth in the UK, on the one hand, and the control condition involving no external information about inequality, on the other.^2^

The individual condition plays two key roles in our design. First, it lets us compare individual participants’ descriptive perception of inequality as measured through our Lego bricks tasks with their answers to items about this perception in our pre-interview survey. This makes it possible to evaluate whether a more informed public—participants introduced to the notions of income, wealth, concentration, and distribution—expresses a different sense of inequality’s extent from that of relatively uninformed survey respondents. Second, comparing responses to inequality items in our post-individual interview and post-focus group surveys enables us to measure the effect of group discussions and interactions on people’s inequality beliefs. We also track this effect by comparing the outputs of our Lego bricks tasks for individual and focus group participants, as well as by analyzing participants’ discourse in our individual and focus group conditions.

### Deliberation in Action: Findings from a Proof-of-Concept Pilot Study in London

In this section we illustrate the contributions of our research design by reflecting on the findings from a pilot study we conducted in the fall of 2019. This study involved three deliberative focus groups of seven to eight participants each, all of whom were residents of the London metropolitan area and had been recruited via an external research recruitment company’s maintained database of research participants, which individuals are invited to join via multiple online and offline channels. All three focus groups were diverse in terms of participants’ socioeconomic background; we did not test the socially homogeneous condition during this study. Each group was assigned to a different information condition: the “true distribution,” “historical data,” and “social mobility” conditions we described earlier.

Because we only collected a limited number of pre- and post-focus group surveys in each condition, and because our pilot did not include any individual interviews, our data do not lend themselves to a quantitative analysis of the impact of deliberation, or of participants’ exposure to different informational treatments, on their understandings of inequality. The small scale of our qualitative material likewise precludes a robust analysis of how deliberation and interactions shine light on the content of participants’ thinking about inequality. Instead, we use the qualitative data from our pilot to showcase the effectiveness of our design for eliciting meaningful social dynamics around the discussion of inequality, and for making participants engage with the idea of distribution, rather than other ideas of inequality.

Pilot focus groups were audio recorded and transcribed intelligent verbatim. During focus group sessions, researchers (the moderator and one or two observers) also kept written observational records which we use as supporting documentation to inform our qualitative analysis. Focus group transcripts were iteratively hand-coded following two main avenues: one was to code thematically for the manifest content of the focus groups, taking an inductive approach; the other was to code for specific elements of group interaction, taking a discursive analytical focus.

#### Situated Talk of Income and Wealth

Our design is motivated by the idea that beliefs about economic inequality have a socially situated character: to the extent that people think and talk about income or wealth inequality in social life, they do so as members of discussion and interaction networks. Our pilot study illustrates how deliberative focus groups recreate these interactional contexts as places for the articulation of inequality beliefs. For example, the following extracts show participants’ responses when we asked them to elaborate on their understandings of “income” and “wealth” near the beginning of our first focus group:

*Moderator*: We’re going to start by thinking about what comes into your head when you hear the word “income”?

*M (Man participant)*: Money.

*M: Investments.*

*W (Woman participant): Jobs.*

*M: Whether it includes tax or not.*

*W: The flow of something inward. Income, something coming in. It could be multiple, single.*

*W: Feeling happy about the bank balance.*

*M: Different sources, whether it’s a job or investments or partner, friends, family.*

*M: Personal company income.*

*M: In my position* [this participant is a student]*, pressure, pressure on people like me, people my age, to find a job, get an income.*

*W: Status, people’s reputation and status, your income can reflect that.*

*M: Relativity, a high income in some positions is not actually that high compared to...*

*(...)*

*Moderator: Did pressure and status resonate with other people?*

*M: Yes.*

*M: Peer pressure, or where I stand with my friends, or even younger relatives. You always think, ‘Maybe they’re doing better than me.’ You always put yourself where you should be at this age, this point in life.*

*(...)*

*M: From personal experience, your income can be like a roller-coaster. One minute you can be very low, and then it can change, upwards and downwards. It’s not static or constant.*

*(...)*

*W: A lot of people assume that it’s going to be stable and it’s going to go on an upward trajectory. Then something happens, and all of a sudden, it’s ripped away from you. You can join a company, and if the policy changes from final salary to a new pension, just because of the day you’ve signed your contract.*

(Focus Group One)

*Moderator: A related word, what do you think of when you hear the word “wealth”?*

*W: Generational.*

*W: Assets.*

*M: Preservation.*

*M: People working less for it, it’s more something that people have.*

*W: It’s something that is nurtured, and continues to build and grow, is passed down.*

*W: You can build your wealth, so someone with the same income might end up with more or less, depending on how they spend it.*

*(...)*

*M: Wealth isn’t necessarily about financial value. You can have a wealth of knowledge or a wealth of experience. It might have some inherent value, but it’s just in terms of demands.*

*W: Assets aren’t necessarily financial.*

(Focus Group One)

From a discursive analytical perspective, this exchange reveals how participants’ expressions of what “income” or “wealth” meant to them were activated by the contributions of other group members. For example, the notion of “pressure” introduced by a student participant resonates with several others who had not brought it up in the first place: a woman participant shifts to a clearer articulation of pressure as related to social standing (“how income can reflect status”). She is quickly seconded by another group member noting how income, as a marker of social position, is relative in nature. This is then built upon by a fourth participant: *“You always think: ‘Maybe they’re doing better than me.’ You always put yourself where you should be at this age, this point in life.”*

Participants’ contributions thus scaffold upon one another as they develop their articulations of income and wealth. We can consider this further through another example where participants connected meanings of income to their own social position and ensuing social perspective:

*Moderator: I’d like to think about, when you hear the word “income,” what comes into your head?*

*W: Salary.*

*W: Everything that comes into your bank.*

*M: Lifestyle.*

*M: Disposable income.*

*M: Taxes.*

*W: What’s going to be left over once all my bills are paid out.*

*W: As a student, I’d say bursary.*

*(...)*

*M: Balance, from the perspective of being a father. My older siblings were discussing with me how much disposable income they had before they became parents. Now we’re all, “Haven’t got the money for that anymore.” Life balance, but certainly the finance side as well, or lack of it.*

(Focus Group Three)

Following more generic statements (“salary,” “everything that comes into your bank”), one participant here weighs in with a definition of income that is overtly dependent upon personal experience (“As a student, I’d say bursary”). This introduction of biographical detail gives others ‘permission’ to draw on aspects of their own lives to make sense of the matter at hand (“Balance, from the perspective of a father”). A similar dynamic gradually centering the relevance of personal experience was also present in our earlier excerpt from focus group one: “in my position [as a student], pressure, pressure on people like me to find a job, get an income,” “from personal experience, your income can be like a roller-coaster.”

These exchanges evidence how individual articulations of income or wealth are contingent on the interactional context created by our deliberative focus groups. Using focus groups recreates these interactions and makes them visible to the researcher.

#### Deliberating Distributions

We now consider what was achieved by incorporating the Lego bricks task. Researchers have used a variety of methodologies to study perceptions of inequality’s extent. Some of these do not directly engage with the notion of distribution. For example, the 2009 ISSP survey includes a common battery of questions asking respondents how much they think people in certain professions—a doctor in general practice, the chairperson of a large national corporation, a shop assistant, an unskilled worker in a factory, and a cabinet minister in the national government—actually earn (e.g., Osberg & Smeeding, 2006; Koçer and van de Werfhorst 2012a, 2012b).^3^ Kiatpongsan and Norton (2014) likewise measure perceptions of inequality by comparing respondents’ estimates of the wages of CEOs and unskilled workers. While this approach has the upside of evoking precise occupations that respondents can relate to, it does not ask them to make distributional choices: they may think of their wage estimates separately, without reflecting on how an overall amount of income is spread out over the income hierarchy. To the extent that people do think about inequality as an unequal repartition of resources in a population, this strategy is unlikely to capture their thinking.

A different approach asks survey respondents to estimate the share of a country’s income going to each of this country’s income quintile or decile (e.g., Alesina et al., 2018; Cruces et al., 2013; Karadja et al., 2017; Norton & Ariely, 2011). This is a straightforward distributional task, yet it is also a cognitively challenging one when executed abstractly, leading respondents to rely on anchoring-and-adjustment heuristics that distort the reporting of their inequality perceptions (Campos-Chambers et al., 2014; Eriksson & Simpson, 2012; Vazquez et al., 2020).^4^

Our Lego bricks activity strives to overcome these issues by asking participants to distribute a fixed amount of Lego pieces over ten income groups. In effect, this forces participants to make distributional choices, confronting them with the zero-sum character of income or wealth at a given time. In addition, the tactile nature of the Lego task, as well as the number of Lego bricks to be allocated (one hundred, which easy translates into percentages of a country’s total income or wealth going to each income or wealth decile) are meant to make the concept of distribution more immediately intuitive, minimizing the need for examples that may work as anchor points.

**Fig. 1:**
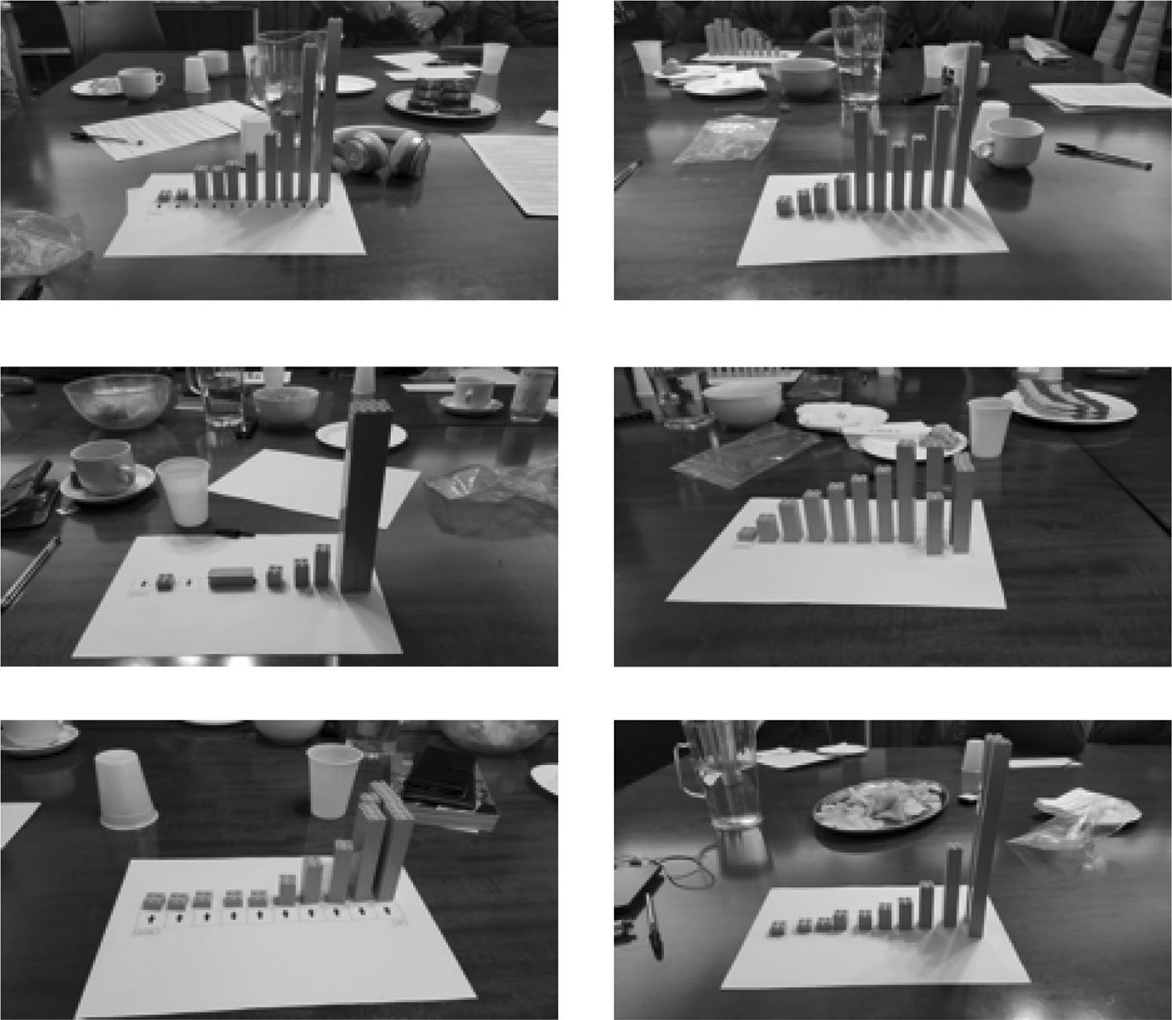
Brick renderings of participants’ estimated UK income distribution in the six subgroups of our second and third focus groups

**Fig. 2:**
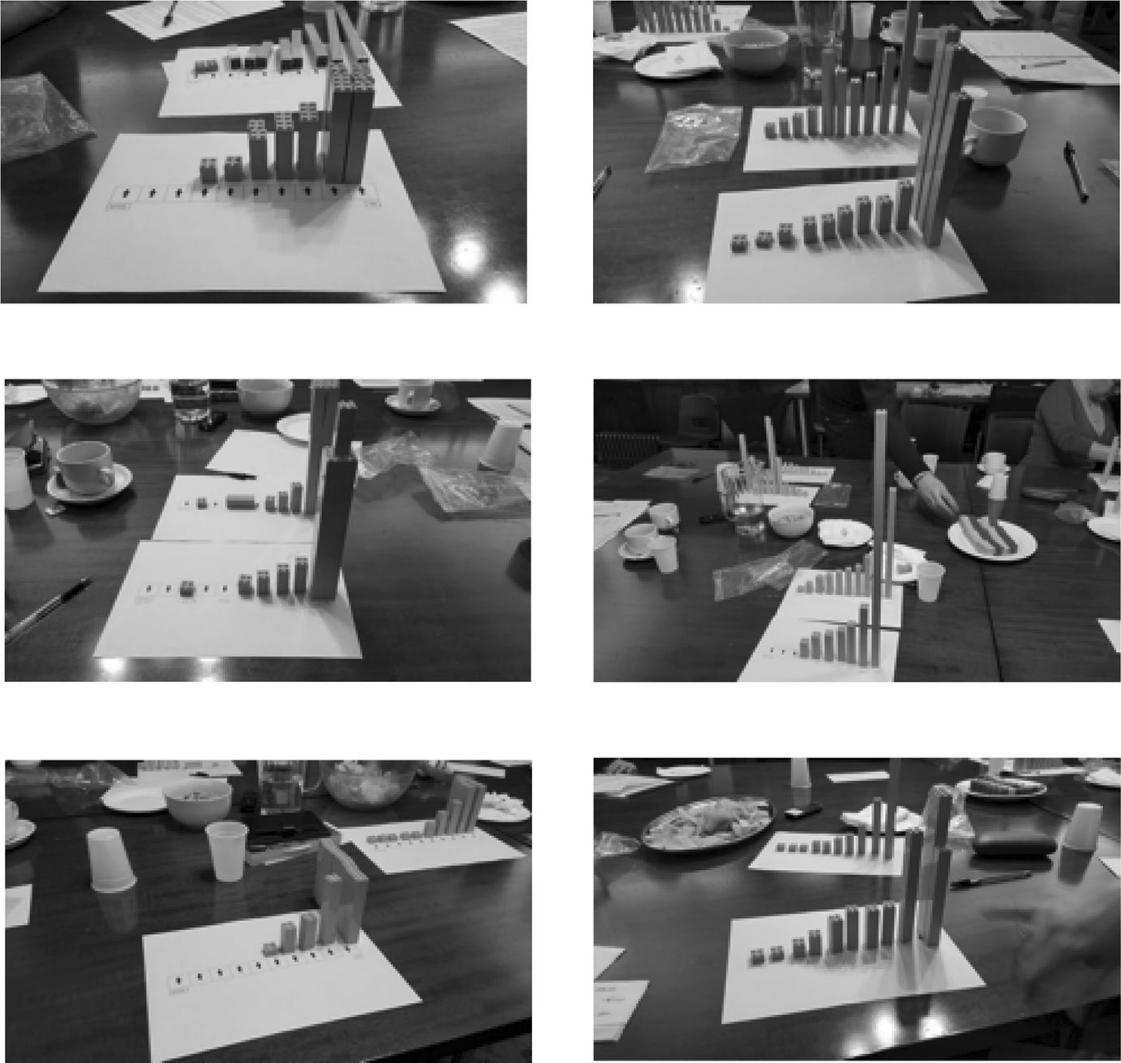
Brick renderings of participants’ estimated UK wealth distribution (in the foreground) in the six subgroups of our second and third focus groups

**Fig. 3:**
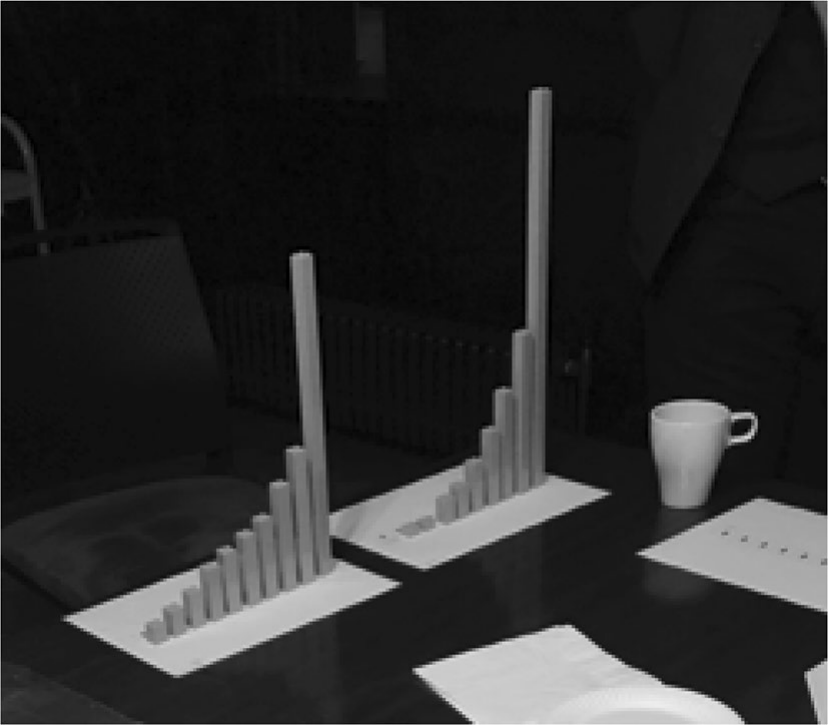
Brick renderings of the actual distributions of income (left) and wealth (right) in the UK today

Figure 1 shows estimations of the UK income distributions made by each group of participants in our pilot’s second and third focus groups. Figure 2 does the same for their estimations of the UK wealth distribution. For comparison, Fig. 3 shows brick renderings of the actual distributions of income and wealth in the UK today based on the World Inequality Database and Wealth and Assets Survey, respectively. We purposely keep these figures low-quality to avoid presenting them as definitive findings, yet we include them to showcase the sort of empirical material our research instrument generates.

Participants in our pilot study quickly became engrossed with the task, as the physical manipulation of bricks brought a play-like activity to the focus groups. They typically began by spreading the bricks out across the table in front of them, before discussing and deciding how to organize them. Rarely, groups wrote down notes or calculations before arranging their bricks. More commonly, they started assembling bricks into stacks and then continued their discussion and moved bricks between the different stacks until their adjustments “looked about right.” Importantly for our purposes, participants appeared to be comfortable with the task’s distributional character. Few asked for clarification as to what the bricks represented. Thinking about bricks in terms of percentages of the overall income or wealth in the UK seemed relatively effortless. For example, here is how one subgroup reported going about the wealth task in our second focus group:

*W: We went for “the wealth is concentrated in the top 10 percent” and we increased it, so we’ve made this 85 percent, so we had 15 percent* [left] *to share. We felt that the two bottom squares* [i.e. deciles] *were people who have no wealth, that if the washing machine broke down . . .*

*M: They’re washing by hand.*

*W: Yes but not a spare penny. (. . .) The folks in this bit* [higher up] *we’ve divided one brick between three of them* [three deciles]*. (. . .) Again it was all concentrated at this end.*

(Focus Group Two, Subgroup One)

The tactile nature of the task meant that participants did not have difficulty thinking of economic inequality in terms of greater or lesser resource concentration (“the wealth is concentrated in the top 10 percent,” “we’ve divided one brick between three of them”). To the extent that they found the task challenging, it was because participants struggled with the substantive decisions they needed to make. As a consequence, the task provided an even clearer impetus for group discussion and deliberation, in line with our methodological focus on inequality perceptions as situated in interaction. Participants did not make decisions quickly, with a tendency for some hesitation at the beginning of the task, followed by thoughtful deliberation—both verbally and through the physical manipulation of bricks—as to how to shape distributions. This sometimes resulted in insights that got them closer to the empirical distribution they were looking to approximate. The next excerpt can thus be compared to Fig. 3 above, which indeed reveals that in the UK, wealth is more concentrated than income:

*M: We were debating that it was the whole UK population, not everybody earns income. It’s biased towards the top 10 percent of the population, and then it gradually tapers down. The wealth is biased* [that is, more heavily biased] *towards the 20 percent, 30 percent of the population. The middle, there’s income there. We did have a debate about income and wealth. If it was the wealth, it would look even more biased towards there* [the top]*. We suppressed it a bit, differentiated between income and wealth.*

(Focus Group One, Subgroup Two).

#### Heuristics of Distribution: Social Groupings, Imagined Lifestyles and Sensing

Our design further aims to capture the qualitative understandings that might animate participants’ execution of our task, and how these might enable or constrain their estimations of inequality. The thematic coding of our pilot’s qualitative material identified three, non-mutually exclusive themes or heuristics in the thinking of participants as they went about allocating the Lego bricks. The first was “social groupings,” whereby participants identified broad social categories, such as classes or occupational groupings, to make sense of different points along the income or wealth distributions and then decide how much to allocate to each group. The second heuristic, “imagined lifestyles,” describes participants’ attempts to envision living standards at various levels of income or wealth in order to make judgments about the distribution. The third heuristic, “sensing, not summing,” describes how participants tended to arrange and rearrange the bricks until they “looked right” to them, instead of engaging in more specific formulas.

To illustrate, here is how one subgroup went about the income task in our first focus group, gradually shifting from the precise allocation of percentages to heuristic groupings of “the richest” vs. “those on minimum wage” as the conversation developed:

*Moderator: We’re going to start thinking about income. What you have are 10 people arranged in a line. These 10 people represent the whole UK population. They’re arranged in order of income. You’re also going to have 100 Lego bricks, representing all of the income in the UK. What I’d like you to do is to allocate these bricks to these people in this line. Think in terms of percentages.*

*M: If we’re talking about income, I would imagine 30 percent there.*

*M: I think it will be heavily concentrated up there.*

*W: Yes, I’m just thinking about the percentage.*

*W: I don’t know, it’s quite hard to gauge.*

*M: Most of it’s sitting . . .* [gestures toward the top deciles]

*(...)*

*M: Shall we just have a lot on this side, the richest people there. What is the share of 100 that they get?*

*M: If the definition of income was just based on what you get from work...*

*M: These* [gesturing toward the bottom deciles] *are going to be the minimum wage.*

*(...)*

*M: There, you’re talking minimum wage, and then slightly up.*

*M: Is what you’re saying that everyone here* [in the bottom deciles] *would just have one block?*

*F: Yes.*

(Focus Group One, Subgroup One).

When deploying “social groupings,” participants appeared to tend to find it more straightforward to imagine groups toward the top of the income or wealth hierarchy. It was striking that this heuristic was not as readily available further down the distribution, making allocation decisions more challenging:

*Moderator: And then this group over here, talk us through how you decided to arrange your bricks like that.*

*W: I mean, initially, we said obviously the top 10 percent such as the CEOs, politicians, and footballers were all quite high. There’s not much of a difference between the top two* [deciles] *but then there is kind of a drop that goes down into more high earners that aren’t at the top of the game. We then had quite a bit of a dip between high earners and the working class. (. . .) And I think we said that the working class, because obviously there are different levels of the working class as well (. . .)*

*Moderator: And you said you found it a bit more difficult to do? Was that because you disagreed?*

*M: There’s just a lot to it to try and think about.*

*M: You kind of already know about the high earners. Because that kind of statistics is always in the media that this percentage earns this amount in the country.*

*Moderator: But what, further down it’s more difficult?*

*M: Yes, that’s what we struggled with.*

(Focus Group Two, Subgroup Two).

The extracts below further highlight how two groups used a similar, “imagined lifestyles” heuristic of adding up various archetypical items (a car, a house, savings, investments) in order to complete our wealth task. While these are but a few examples, they showcase the potential of our research instrument for reveal fine-grained logics behind people’s thinking about income or wealth distributions:

*W: As we got here* [the sixth decile] *we felt this was the 50 percent of the population who probably own their own house but probably because they’re of an age where they were able to get onto the property market, which now is much harder unless you have the bank of mum and dad to help you. We get these other ones* [higher up]*, these represented people who had a house, a car and some savings. This* [higher up again] *was if you had a house, car, savings and shares, whether they’ve come to you via the company you work for and this one* [higher still] *was you had the house, the car, the savings, your stocks and shares, and maybe a bit of investment. Again, it was all concentrated at this end* [the top decile]*.*

(Focus Group Two, Subgroup One).

*W: We started off by thinking of the unemployed, they definitely don’t have any wealth but it’s unlikely for them to have any assets, like cars and properties. The working class might have bought a car, they maybe have property. From there about six up we started saying, “Okay, people are more likely to have a car and maybe be on the property ladder.” As we get to the top end that’s when they have a lot more assets, the houses are expensive, they have more stocks, shares. It's bigger.*

(Focus Group Two, Subgroup Two).

Finally, our pilot demonstrated how the bricks task itself was a vehicle for the sharing of perspectives among participants and the collective formation of inequality beliefs. Focus group members commonly let the insights of others alter their own understandings of inequality, for example when participants introduced the idea that economic disparities in London might be more pronounced than in other parts of the country, or when they shared information about inequality they had heard from the media. Participants also signaled their willingness to defer to others on aspects of the distribution they felt less familiar with:

*W: I found* [the task] *quite difficult because it’s such a broad look at the UK. There are some demographics that I don’t come into contact with. (. . .) One thing I would do now, looking at the other guys’ graphs, is take this away* [that is, bricks at the bottom of her subgroup’s distribution] *because when you’re on the breadline, you don’t have anything. I don’t know much about benefits, but it’s nothing.*

(Focus Group One)

Because all of our pilot focus groups were socioeconomically diverse, it remains an empirical question whether these deliberative dynamics would play out differently in more homogeneous groups, and whether diverse groups arrive at more accurate estimates of income or wealth inequality than do homogeneous ones or isolated individuals. This article’s only empirical ambition was to demonstrate the power of deliberative focus groups, incorporating a tactile task, as a research instrument for eliciting meaningful, discursive thinking about economic inequality.

## Conclusion

Starting from the premise that individuals form beliefs about economic inequality through the way they experience the local worlds they are embedded in, this article has proposed a blueprint for studying the role of discussion and interaction networks in shaping people’s understandings of inequality. The methodology of deliberative focus groups we advocate aims to approximate these networks, and contrast them with artificially diverse networks, while also letting us explore whether different types of information circulating within them result in the formation and articulation of different inequality beliefs. Ultimately, this approach should enable us to test a number of important outstanding questions about descriptive and normative understandings of inequality, such as whether the low socioeconomic diversity of people’s discussion and interaction networks is responsible for their tendency to underestimate it, or whether beliefs in opportunity explain low public support for taxation and redistribution.

Because of public health restrictions during the Covid-19 pandemic, at this stage our blueprint remains just that. We strived to demonstrate its promise, however, by showcasing the effectiveness of deliberative focus groups in recreating the kind of interactional dynamics our project is after. The effects of these dynamics we hope to observe in the not-too-distant future, when people start talking in person again – about inequality and other matters.

## Supplementary Information

Below is the link to the electronic supplementary material.

Supplementary file1 (DOCX 114 kb)

## Footnotes

1 In our pilot study, all participants hailed from the London metropolitan area, meaning that they all shared the experience of living in a densely populated and highly unequal setting.

2 This means that our full design has 14 conditions total: Three deliberative focus group conditions (one diverse, two homogeneous) x four information conditions (“true distribution,” “historical data,” “social mobility,” and “no information”), plus one individual condition x two information conditions (“true distribution” and “no information”). The pre- and post-interview surveys taken by participants in this condition are the same as those in our deliberative focus groups.

3 The ISSP questions are also followed by a similar battery of items asking respondents what they think each of these workers *should* earn.

4 For example, Hecht (2021) finds that even among highly educated top income earners in the UK, some participants had difficulty with the concept of distribution as zero-sum at a point in time. Norton and Ariely (2011) made participants estimate the actual distribution of wealth in the US by asking them to indicate what percentage of wealth they thought was owned by each of five wealth quintiles, starting with the top 20% and ending with the bottom 20%. To help with this task, the authors offered participants two extreme examples, instructing them to assign 20% of the wealth to each quintile if they thought each quintile had the same amount of wealth, and 100% of the wealth to one quintile if they thought one quintile concentrated all of it. Eriksson and Simpson (2012) show how the first example worked as an anchor for participants’ estimates, biasing the authors’ measure of inequality perceptions downwards.

## Declarations

### Availability of data and material

Due to the sensitive nature of the focus group conversations, for reasons of privacy, recordings or transcripts are not made available.
