# Supplementary material for: Deliberating Inequality: A Blueprint for Studying the Social Formation of Beliefs about Economic Inequality
Source: Soc Justice Res. 2022 Apr 1;35(4):379–400. doi: 10.1007/s11211-022-00389-0 (PMC8972749; doi:10.1007/s11211-022-00389-0)
Supplement: Supplementary file 1 — Supplementary file1 (DOCX 114 kb) [file 11211_2022_389_MOESM1_ESM.docx]

**Appendix A: Survey Questionnaires**

*1. Pre-Focus Group Survey*

**London School of Economics, Wealth and Income in Society**

Before we start our discussion, we would like to ask you a few questions about our topic today. These questions have no right or wrong answers: we just ask that you answer them individually and to the best of your abilities. Please take your time.

1. To begin we have some questions about opportunities for getting ahead. First, we would like to know how you feel about the following statement:

“In the UK everyone has a chance to make it and be economically successful.”

| 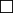 | 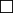 | 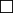 | 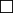 | 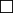 |
| --- | --- | --- | --- | --- |
| Strongly agree | Agree | Neither agree nor disagree | Disagree | Strongly disagree |

2. Next, we would like to know what you think people in these jobs actually earn. Please write in how much you think they usually earn each year, before taxes. Many people are not exactly sure about this, but your best guess will be close enough. This may be difficult, but it is very important. So please try.

1. About how much do you think a doctor in general practice earns?

…………………………£ per year

1. How much do you think a chairperson of a large national company earns?

…………………………£ per year

1. How much do you think a shop assistant earns?

…………………………£ per year

1. How much do you think an unskilled worker in a factory earns?

…………………………£ per year

1. How much do you think a cabinet minister in the national government earns?

…………………………£ per year

3. Now, we would like to know what you think people in these jobs should earn each year (before taxes), regardless of what they actually get.

1. About how much do you think a doctor in general practice should earn?

…………………………£ per year

1. How much do you think a chairperson of a large national company should earn?

…………………………£ per year

1. How much do you think a shop assistant should earn?

…………………………£ per year

1. How much do you think an unskilled worker in a factory should earn?

…………………………£ per year

1. How much do you think a cabinet minister in the national government should earn?

…………………………£ per year

4. Do you think that the very high earners in our society deserve their high incomes?

| 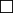 | 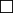 | 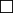 |
| --- | --- | --- |
| Most of the time | Sometimes | Rarely |

5. To what extent do you agree or disagree with the following statements?

“Differences in income in the United Kingdom are too large.”

| 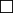 | 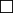 | 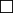 | 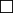 | 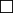 |
| --- | --- | --- | --- | --- |
| Strongly agree | Agree | Neither agree nor disagree | Disagree | Strongly disagree |

“Differences in wealth in the United Kingdom are too large.”

| 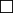 | 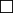 | 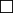 | 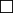 | 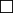 |
| --- | --- | --- | --- | --- |
| Strongly agree | Agree | Neither agree nor disagree | Disagree | Strongly disagree |

6. Do you think it is possible nowadays to reduce differences in income between people with high incomes and people with low incomes?

| 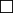 | 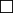 | 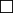 | 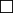 | 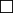 |
| --- | --- | --- | --- | --- |
| Definitely possible | Difficult but possible | Almost impossible | Not possible  at all | Differences in income do not need to be reduced |

7. Do you think it is possible nowadays to reduce differences in wealth between people with much wealth and people with little wealth?

| 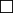 | 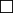 | 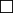 | 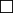 | 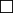 |
| --- | --- | --- | --- | --- |
| Definitely possible | Difficult but possible | Almost impossible | Not possible  at all | Differences in income do not need to be reduced |

Finally, we would like to follow up with you in a couple of weeks with another quick survey. We will be sending this second survey through the post. We would therefore like you to write in the address where we are most likely to reach you. Thank you!

My name and address:

…………………………………………………..

…………………………………………………..

*2. Post-Focus Group Survey*

**London School of Economics, Wealth and Income in Society**

1. To begin we have some questions about opportunities for getting ahead. First, we would like to know how you feel about the following statement:

“In the UK everyone has a chance to make it and be economically successful.”

| 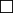 | 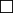 | 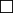 | 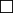 | 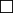 |
| --- | --- | --- | --- | --- |
| Strongly agree | Agree | Neither agree nor disagree | Disagree | Strongly disagree |

2. Now, we would like you to tick one box for each of these to show how important you think it is for getting ahead in life…

1. Coming from a wealthy family?

| 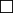 | 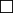 | 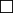 | 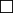 | 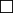 |
| --- | --- | --- | --- | --- |
| Essential | Very important | Fairly important | Not very important | Not important  at all |

1. Having well educated parents?

| 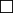 | 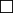 | 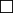 | 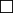 | 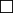 |
| --- | --- | --- | --- | --- |
| Essential | Very important | Fairly important | Not very important | Not important  at all |

1. Having a good education yourself?

| 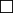 | 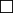 | 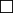 | 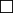 | 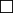 |
| --- | --- | --- | --- | --- |
| Essential | Very important | Fairly important | Not very important | Not important  at all |

1. Hard work?

| 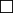 | 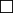 | 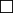 | 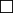 | 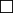 |
| --- | --- | --- | --- | --- |
| Essential | Very important | Fairly important | Not very important | Not important  at all |

1. Knowing the right people?

| 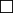 | 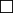 | 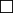 | 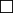 | 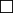 |
| --- | --- | --- | --- | --- |
| Essential | Very important | Fairly important | Not very important | Not important  at all |

1. Having political connections?

| 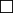 | 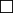 | 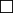 | 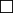 | 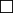 |
| --- | --- | --- | --- | --- |
| Essential | Very important | Fairly important | Not very important | Not important  at all |

1. Giving bribes?

| 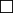 | 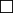 | 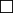 | 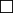 | 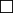 |
| --- | --- | --- | --- | --- |
| Essential | Very important | Fairly important | Not very important | Not important  at all |

1. A person’s ethnicity?

| 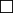 | 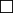 | 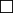 | 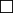 | 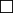 |
| --- | --- | --- | --- | --- |
| Essential | Very important | Fairly important | Not very important | Not important  at all |

1. A person’s religion?

| 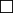 | 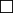 | 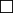 | 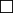 | 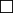 |
| --- | --- | --- | --- | --- |
| Essential | Very important | Fairly important | Not very important | Not important  at all |

1. Being born a man or a woman?

| 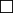 | 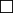 | 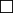 | 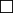 | 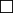 |
| --- | --- | --- | --- | --- |
| Essential | Very important | Fairly important | Not very important | Not important  at all |

3. Next, we would like to know what you think people in these jobs actually earn. Please write in how much you think they usually earn each year, before taxes. Many people are not exactly sure about this, but your best guess will be close enough. This may be difficult, but it is very important. So please try.

1. About how much do you think a doctor in general practice earns?

…………………………£ per year

1. How much do you think a chairperson of a large national company earns?

…………………………£ per year

1. How much do you think a shop assistant earns?

…………………………£ per year

1. How much do you think an unskilled worker in a factory earns?

…………………………£ per year

1. How much do you think a cabinet minister in the national government earns?

…………………………£ per year

4. Now, we would like to know what you think people in these jobs should earn each year (before taxes), regardless of what they actually get.

1. About how much do you think a doctor in general practice should earn?

…………………………£ per year

1. How much do you think a chairperson of a large national company should earn?

…………………………£ per year

1. How much do you think a shop assistant should earn?

…………………………£ per year

1. How much do you think an unskilled worker in a factory should earn?

…………………………£ per year

1. How much do you think a cabinet minister in the national government should earn?

…………………………£ per year

5. Do you think that the very high earners in our society deserve their high incomes?

| 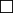 | 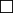 | 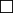 |
| --- | --- | --- |
| Most of the time | Sometimes | Rarely |

6. Next, we would like to ask you to think about wealth, including savings, financial assets, and – if owner-occupied – the value of one’s home(s). Please write in how much wealth (in £) you think people in the following jobs typically have. We understand that it varies, but think of what the middle of the range for someone in each type of occupation might be.

1. About how much wealth do you think a doctor in general practice has?

…………………………£

1. How much wealth do you think a chairperson of a large national company has?

…………………………£

1. How much wealth do you think a shop assistant has?

…………………………£

1. How much wealth do you think an unskilled worker in a factory has?

…………………………£

1. How much wealth do you think a cabinet minister in the national government has?

…………………………£

7. Finally, we would like to know how much wealth (in £) you think people in these jobs should have, regardless of what they actually have.

1. About how much wealth do you think a doctor in general practice should have?

…………………………£

1. How much wealth do you think a chairperson of a large national company should have?

…………………………£

1. How much wealth do you think a shop assistant should have?

…………………………£

1. How much wealth do you think an unskilled worker in a factory should have?

…………………………£

1. How much wealth do you think a cabinet minister in the national government should have?

…………………………£

8. Let’s continue to think about how much people have in life. Below is a description of some key financial facts for 4 different families (families A, B, C, and D). These families each live in the Greater London area and are made up of two parents in their early-forties living with their two children:

**Family A**

House: Social Housing Flat / 45m^2^

Car: None

Savings: None

**Family B**

House: Terraced Rental / 60m^2^ / No garden

Car: Second-hand Ford Fiesta

Savings: Less than £10,000

**Family C**

House: Semi-detached / 80m^2^ / Small garden

Car: Volkswagen Passat

Savings: Around £50,000

**Family D**

House: Detached House / 200m^2^ / Large garden

Cars: Audi A4 and Volkswagen Passat

Savings: Over £500,000

Thinking about all families in the Greater London area with two parents in their early 40s and two children, what percentage of these families do you think live like families A, B, C and D?

1. Percentage of these families who live like family A: . . . . %
2. Percentage of these families who live like family B: . . . . %
3. Percentage of these families who live like family C: . . . . %
4. Percentage of these families who live like family D: . . . . %

9. Do you think inequality is a serious problem in the UK?

| 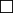 | 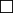 | 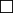 | 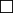 | 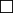 |
| --- | --- | --- | --- | --- |
| Not a problem  at all | A small problem | A problem | A serious problem | A very serious problem |

10. To what extent do you agree or disagree with the following statements?

“Differences in income in the United Kingdom are too large.”

| 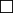 | 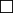 | 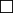 | 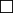 | 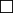 |
| --- | --- | --- | --- | --- |
| Strongly agree | Agree | Neither agree nor disagree | Disagree | Strongly disagree |

“Differences in wealth in the United Kingdom are too large.”

| 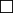 | 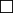 | 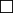 | 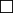 |  |
| --- | --- | --- | --- | --- |
| Strongly agree | Agree | Neither agree nor disagree | Disagree | Strongly disagree |

“It is the responsibility of the government to reduce the differences in income between people with high incomes and those with low incomes.”

|  |  |  |  |  |
| --- | --- | --- | --- | --- |
| Strongly agree | Agree | Neither agree nor disagree | Disagree | Strongly disagree |

“The government should provide a decent standard of living for the unemployed.”

|  |  |  |  |  |
| --- | --- | --- | --- | --- |
| Strongly agree | Agree | Neither agree nor disagree | Disagree | Strongly disagree |

“It is the responsibility of private companies to reduce pay differences between their employees with high pay and those with low pay.”

|  |  |  |  |  |
| --- | --- | --- | --- | --- |
| Strongly agree | Agree | Neither agree nor disagree | Disagree | Strongly disagree |

11. Looking at the list, who do you think should have the greatest responsibility for reducing differences in income between people with high incomes and people with low incomes? (please choose one)

Private companies

Government

Trade unions

High-income individuals themselves

Low-income individuals themselves

Income differences do not need to be reduced

12. Do you think it is possible nowadays to reduce differences in income between people with high incomes and people with low incomes?

|  |  |  |  |  |
| --- | --- | --- | --- | --- |
| Definitely possible | Difficult but possible | Almost impossible | Not possible  at all | Differences in income do not need to be reduced |

13. Do you think it is possible nowadays to reduce differences in wealth between people with much wealth and people with little wealth?

|  |  |  |  |  |
| --- | --- | --- | --- | --- |
| Definitely possible | Difficult but possible | Almost impossible | Not possible  at all | Differences in income do not need to be reduced |

14. Finally, we would like you to think about the United Kingdom today and in the future. The five diagrams below show different types of society. Please read the descriptions and look at the diagrams.

1. What type of society do you think the United Kingdom is today? Which diagram comes closest?

|  |  |  |  |  |
| --- | --- | --- | --- | --- |
| Type A | Type B | Type C | Type D | Type E |

1. Thinking about the future, what type of society do you think the UK will be 25 years from now? Which diagram comes closest?

|  |  |  |  |  |
| --- | --- | --- | --- | --- |
| Type A | Type B | Type C | Type D | Type E |

|  |  |  |
| --- | --- | --- |
| Female | Male | Other |

15. Please select your gender

16. What is your age?

16-24

25-34

35-44

45-54

55-64

65-74

75 or older

17. What is your highest level of educational qualification?

|  |  |  |  |  |
| --- | --- | --- | --- | --- |
| No formal qualifications | GCSE or equivalent | A-level or equivalent | Degree | Postgraduate degree |

18. Are you currently employed, unemployed, retired or a full-time student?

|  |  |  |  |  |  |
| --- | --- | --- | --- | --- | --- |
| Full time | Part time | Self-employed | Not currently employed | Student | Retired |

19. If employed or self-employed, what do you do for a living?

. . . . . . . . . . . . . . . . . . . . . . . . . . . . . . . . . . . . . . . . . . . . . . . . . . . . . . . . . . . . . . . .

20. What is your annual household income, before taxes?

Less than £10,000

£10,000-£19,999

£20,000-£29,999

£30,000-£39,999

£40,000-£49,999

£50,000-£59,999

£60,000-£79,999

£80,000-£99,999

£100,000-£149,999

More than £150,000

Don’t know

Prefer not to say

21. If you had to choose one from this list, which social class would you say you belong to?

|  |  |  |  |
| --- | --- | --- | --- |
| Working class | Lower middle class | Upper middle class | Upper class |

22. What is (or was) the highest level of educational qualification of your father?

|  |  |  |  |  |
| --- | --- | --- | --- | --- |
| No formal qualifications | GCSE or equivalent | A-level or equivalent | Degree | Postgraduate degree |

23. What is (or was) the highest level of educational qualification of your mother**?**

|  |  |  |  |  |
| --- | --- | --- | --- | --- |
| No formal qualifications | GCSE or equivalent | A-level or equivalent | Degree | Postgraduate degree |

24. What political party did you vote for in the last parliamentary elections, if any?

Conservatives

Labour

Liberal Democrats

Other (please write in): . . . . . . . . . . . . . .

Did not vote

Prefer not to say

25. Do you have children and, if so, how many?

. . . . . . . . . .

26. Do you and/or your partner own your home?

|  |  |
| --- | --- |
| Yes | No |

27. Do you have savings amounting to at least 4-months worth of your income?

|  |  |  |
| --- | --- | --- |
| Yes | No | Don’t  know |

28. Do you own stocks, bonds or investments amounting to at least 4-months’ worth of your income?

|  |  |  |
| --- | --- | --- |
| Yes | No | Don’t  know |

**Appendix B: Focus Group Topic Guide**

**Focus group preparation**

*Prepare room – tables and chairs, food and drink*

*Equipment for treatments*

*Surveys and consent forms laid out*

*Recording devices*

**Welcome and introductions**

- Please take a seat.
- Help yourself to food and drink.
- Ensure consent forms are signed and returned.
- In front of you there is a survey, when you are ready please could you fill this in by yourself.

**Administer pre-focus group survey**

- *Short, about 5 min. in length, in silence*

**Warm up**

- Thank you very much for filling out those surveys. As you can probably tell now, today we’re going to be talking about how much different people have in society.
- *Researchers in the room introduce themselves.* My name is [Moderator 1], I’m going to be guiding the discussion today and [Moderator 2] might also have some things they want to ask or talk about later on.
  - *Relevant announcements – length of FG, toilets, exits, etc.*
- We are going to be talking about things that people might have different responses to or views about. I want to emphasise that there are not “right” or “wrong” things that you can say. Outside of this room everyone here has different roles, things they do, things they know about, and so on. Inside this room you are all equally valued participants with valuable things to contribute. You might not necessarily see things the same way as others in this room, and that is fine. We should aim to understand and unpack our views.
- Our focus today is on how much different people have in society.
- If we could begin by going around the group and briefly introducing ourselves. Your name and a boring fact about yourself.
- So first, to get us thinking, what do you think of when you hear the word “income”?
  - *Note key input on flip chart.*
  - *Provide clear definition of income.* “Money you receive from work, financial assets or real estate, social security/benefits (give brief examples)”
- And what about when you hear the word ‘wealth’?
  - *Note key input on flip chart.*
  - *Provide clear definition of wealth.* “The amount of assets that someone owns. This can include their house or houses, cars, savings, stocks and shares, investments, and so on.”

**Baseline discussion**

- So let’s think in a more detail about how much different people have in society. Thinking specifically about the UK today, how do you think income is spread out between people?
- *Hand around equipment for visualization of income and wealth inequality.*
- Let’s begin by working in pairs or threes, and then you will share with the rest of the group what you’ve done.
- In front of you, you’ve got 100 Lego bricks, and on your piece of paper you’ve got 10 people organized in a line. The 10 people represent the whole of the UK population and the bricks represent income. The people are lined up in order from lowest to highest in terms of income. We want you to arrange the bricks to show how you think income is spread out among people in the UK. So for example, if you thought income was totally evenly shared out, everyone would have 10 bricks each.
- *Give small groups time to create representation.*
- Fantastic, so let’s look at what each group has done. Talk us through how you’ve laid this out. Why have you arranged the bricks like this? What was easy or difficult to decide?
- Let’s compare between groups. Are there similarities or differences? Why do you think this is?
- Fantastic, now we’re going to do the same exercise but thinking about wealth now.
- *Same instructions and prompts.*
- And what do you think is the reason behind these patterns you’ve created? What means income/wealth are spread out in this way? Why might someone be *here* on the scale as compared to *here*?
- What do you think are some of the positives or negatives about the way in which income/wealth is distributed in the UK today?
- People have different views about whether the way things currently are is an issue or not. What do you think? Is it an issue the way things are? What could be done to change things? Should something be done?

**Treatment**

- *TREATMENT 1 – Extent of income and wealth inequality*
  - So I’m now going to show you the actual way in which income and wealth is shared in the UK today.
  - *Show true representations using same equipment as used by participants*
  - *Check participant understanding*. How does this differ from you made? Does this surprise you?
- *TREATMENT 2 – Challenging inevitability historically.*
  - *Run through Treatment 1.*
  - *Then show visualizations for historical distributions.*
  - *Check participant understanding*. What’s your first reaction? Does this surprise you?
- *TREATMENT 3 – Social mobility.*
  - *Run through Treatment 1.*
  - *The use the visualizations to show income mobility between parent and child.*
  - *Check participant understanding*. What’s your first reaction? Does this surprise you?
- *TREATMENT 4 – No information, jump to next section.*

**Describing Inequality**

- So let’s revisit some of the things we have been talking about.
- Near the beginning of our discussing we talked about your thoughts on how much different people have in society, what do you think about that now?
  - *Prompt with reference to opening survey responses.*
  - Has anything changed for you compared to earlier in the conversation? Have other things stayed the same? Why is that?
  - *Draw out consensus and disagreement within group and explore.*
- Why do you think some people have higher incomes than others?
  - *Prompts:* What about… coming from a wealthy family?; having well educated parents?; having a good education yourself?; hard work?; knowing the right people?; having political connections?; giving bribes?; a person’s ethnicity?; a person’s religion?; being born a man or a woman?
- And what about wealth, why do some people have higher wealth than others?
  - *Prompts: repeat any relevant prompts from previous income question.*

**Evaluating Inequality**

- Let’s think a bit more about whether how much different people have might be a positive or negative thing.
- How does how much different people have affect society?
  - What might be some of the positive things be? *Reference earlier points of discussion*.
  - What might some of the negative things be?

Do you think income/wealth differences in the UK are too large? Why/why not?

- What might we want to change?
- How might things be changed?
- Is change possible?

**Baseline revisit**

- *Pick up on anything relating to 1) what is inequality; 2) what causes inequality; 3) is (are aspects of) inequality good or bad; 4) what should (could?) be done about it? If not adequately covered in preceding two sections*.

**Cool down**

- Thank you so much for your contributions.
- Do you have anything else to add?
- Is there anything you think I’ve missed or misunderstood? Is there anything I should have asked you about but didn’t?
- Any last questions?
- *Explain follow up survey in 2 weeks – emphasize crucial to fill out.*
